# Supplementary material for: Formation of quantum spin Hall state on Si surface and energy gap scaling with strength of spin orbit coupling
Source: Sci Rep. 2014 Nov 19;4:7102. doi: 10.1038/srep07102 (PMC4236754; doi:10.1038/srep07102)
Supplement: Supplementary Information [file srep07102-s1.doc]

**-Supplementary Information-**

**Formation of quantum spin Hall state on Si surface and energy gap scaling with strength of spin orbit coupling**

*Miao Zhou^1^, Wenmei Ming^1^, Zheng Liu^1^, Zhengfei Wang^1^, Yugui Yao^2^, and Feng Liu^1*^*

*^1^* Department of Materials Science and Engineering, University of Utah, UT 84112

*^2^* School of Physics, Beijing Institute of Technology, Beijing, China 100081

*fliu@eng.utah.edu

**CONTENTS**

1. **Computational Details**
2. **Z_2_ Invariant Calculation Results**
3. **Band Structures of Sb and Sn@H-Si(111)**

**References**

1. **Computational Details**

First-principles electronic structure calculations based on density functional theory (DFT) were carried out using the plane-wave-basis-set and the projector-augmented-wave method, as implemented in the VASP code. The energy cutoff was set to 500 eV. For the exchange and correlation functional, the generalized gradient approximation (GGA) in Perdew-Burke-Ernzerhof (PBE) format was used. Spin-orbit coupling (SOC) is included by a second variational procedure on a fully self-consistent basis.

H-Si(111) surfaces were modeled by using a slab geometry of ten atomic layers, with a vacuum region of 30 Å in the direction normal to the surface. Test calculations were performed by using larger thickness (twelve and sixteen layers) which gave similar results. In the pure H-Si(111) surface, both the top and bottom Si surfaces were terminated by H atoms in a monohydride form. For heavy metal atom (Bi, Pb, Sb, Sn, Ga, In and Tl) deposited H-Si(111), a $\sqrt{3}\times\sqrt{3}$supercell was used with two of the three H atoms removed and re-adsorbed with heavy atoms into a hexagonal symmetry. During structural optimization, both the tenth layer of Si atoms and the H atoms saturating them were fixed and all other atoms were fully relaxed until the atomic forces were smaller than 0.01 eV/Å. A 15×15×1 *Γ*-centered *k*-point mesh was used to sample the Brillouin zone. Dipole corrections were also tested and found making little difference.

Z_2_ invariant calculations were performed by using the program package WIEN2K. We employed the full-potential linearized augmented plane-wave method^2^ within the GGA-PBE functional including SOC. A converged ground state was obtained using 5000 k-points in the first Brillouin zone and *K*_max_ × *R*_MT_ = 8.0, where *K*_max_ is the maximum size of the reciprocal lattice vectors and *R*_MT_ denotes the muffin-tin radius. Wave functions and potentials inside the atomic sphere are expanded in spherical harmonics up to *l* = 10 and 4, respectively. For Z_2_ calculation, we follow the method by Fukui *et al.*^2^, to directly perform the lattice computation of the Z_2_ invariants from first-principles, of which the detailed methodology is presented in the Refs. [21] and [36] of the paper. Here we present the calculated Z_2_ number of Bi/Pb@H-Si(111), planar Bi hexagonal lattice, and Bi lattice with one side saturated by H, in Figs. S1-S4, respectively.

Furthermore, effective tight-binding (TB) Hamiltonians of the four-band model involving *p_x_* and *p_y_* orbitals in a hexagonal lattice with SOC are constructed (Eq. 1 in the paper) to qualitatively understand the SOC effect on the band gap opening mechanism. The results are shown in Fig. 4, from which we can see a gradual change of different gap sizes leading to a shift of global gap from *K* to *Γ* point, which correctly reproduces the DFT results of Pb@H-Si(111) and Bi@H-Si(111), as shown in Figs. 2(d) and (c) in the paper.

1. **Z_2_ Invariant Calculation Results**


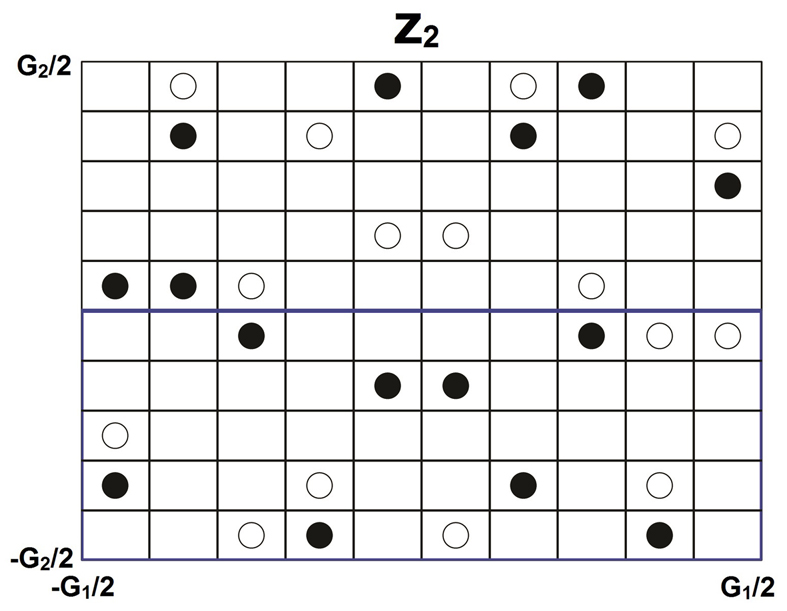


Fig. S1: The n-field configuration for Bi@H-Si(111). The calculated torus in Brillouin zone is spanned by G_1_ and G_2_ (Note that the two reciprocal lattice vectors form an angle of 120°). The solid dots and open circles denote n = 1 and −1, respectively, and the blank denotes 0. The Z_2_ invariant is calculated by summing the n fields over half of the tori, which gives Z_2_ = 1 indicating a topological insulator, in agreement with the edge state calculations [Fig. 3(b)].


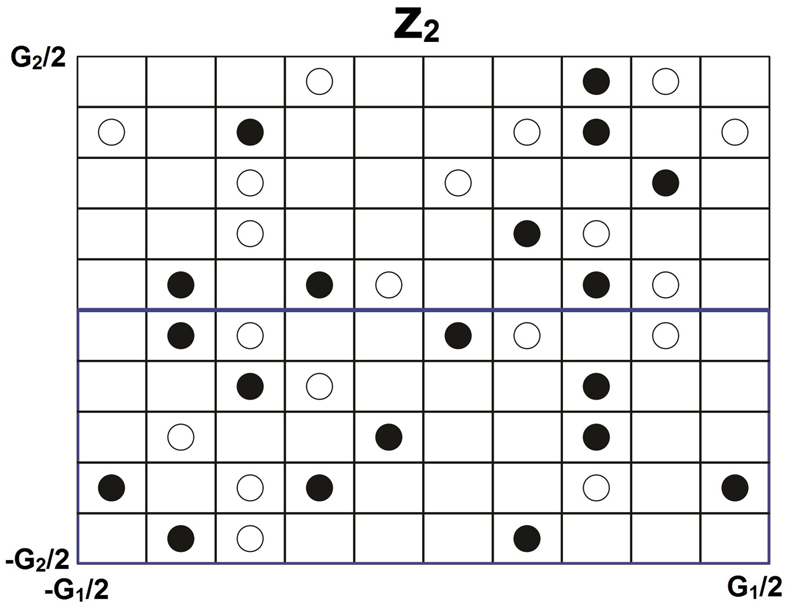


Fig. S2: Same as FIG. S1 for Pb@H-Si(111), by assuming a shift of Fermi level above the lower branch of Dirac band, as shown in Fig. 2 (e). Z_2_ = 1, indicating a topological insulator.


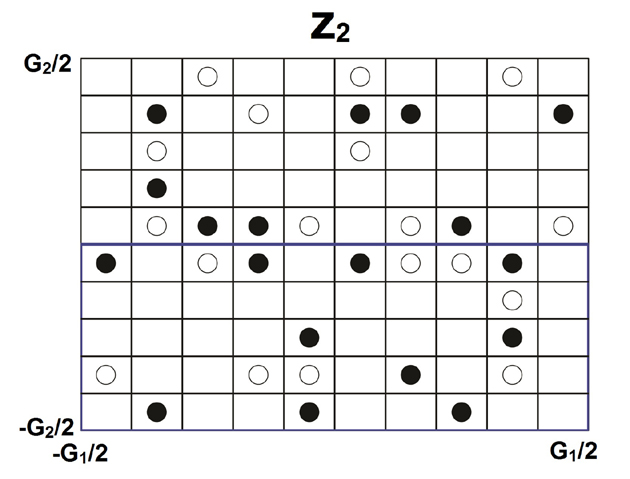


Fig. S3: Same as FIG. S1 for the planar hexagonal lattice of Bi. Z_2_ = 0, indicating a trivial insulator.


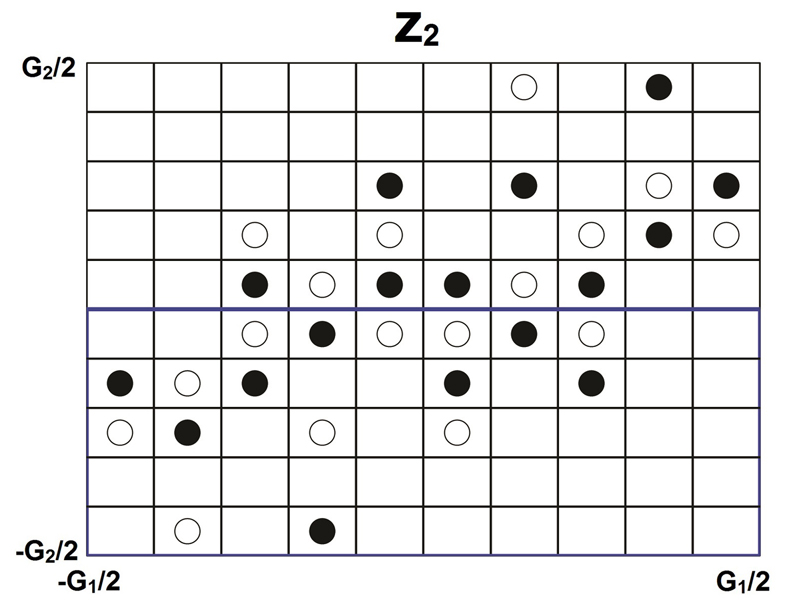


Fig. S4: Same as FIG. S1 for the planar Bi hexagonal lattice with one side saturated by H. Z_2_ =1, indicating a topological insulator

1. **Band structures of Sb and Sn@H-Si(111)**


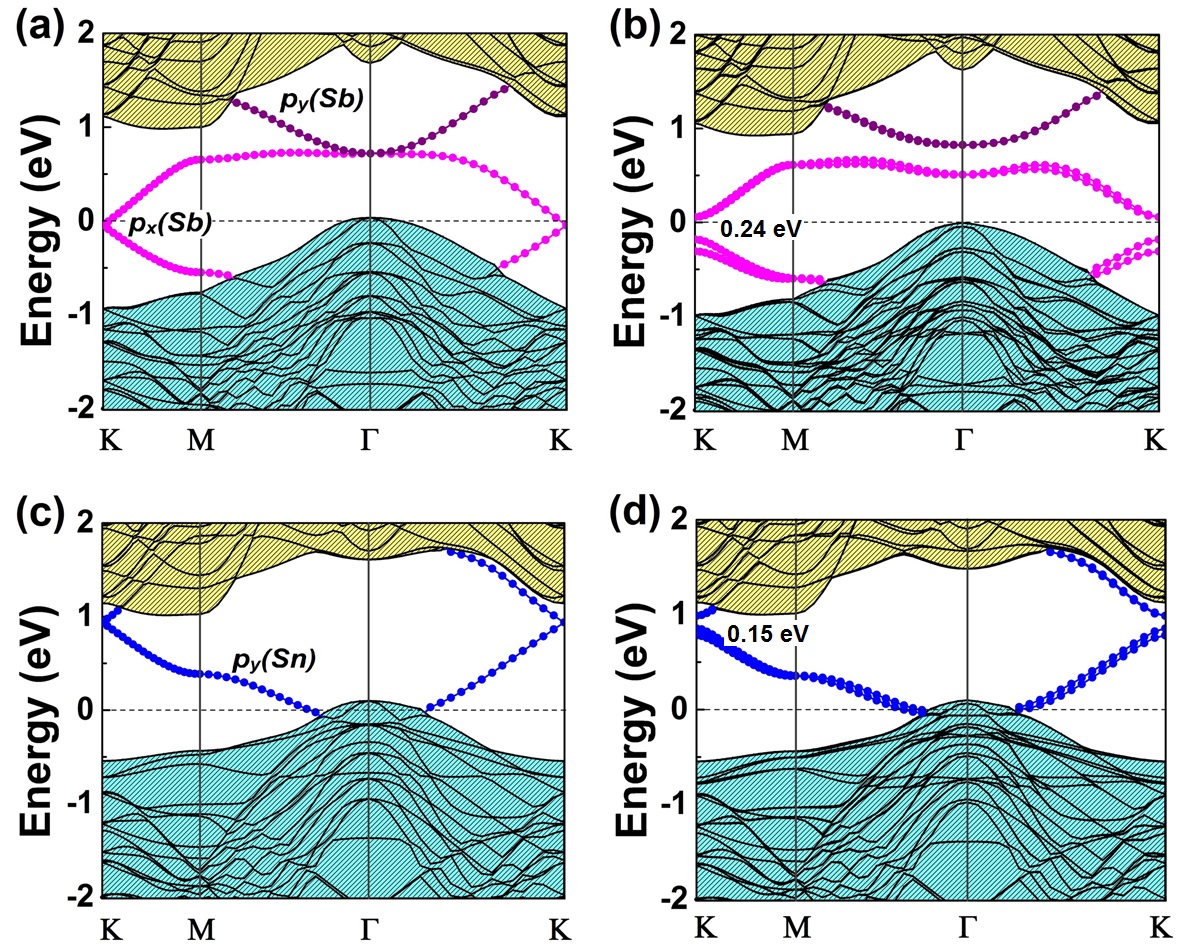


Fig. S5: (a-b) Band structures of Sb@H-Si(111) without and with SOC, respectively. The Fermi level is set to zero. (c-d) Same as (a-b) for Sn@H-Si(111). Band compositions around the Fermi level are indicated in (a) and (c), and the SOC induced energy gaps are indicated in (b) and (d).

**Reference**

[1] Singh, D. J. & Nordstrom, L. Planewaves, Pseudopotentials and the LAPW Method (Kluwer Academic, Boston, 1994).

[2] Fukiu, T. & Hatsugai, Y. Quantum spin Hall effect in three dimensional materials: Lattice computation of Z_2_ topological invariants and its application to Bi and Pb. *J. Phys. Soc. Jpn.* **76**, 053702 (2007).
